# Overexpression of *GmHIR1* in Soybean Enhances *Phytophthora sojae* Resistance

**DOI:** 10.3390/plants15142211

**Published:** 2026-07-20

**Authors:** Zhenyu Xu, Yuecheng Tang, Haishun Ru, Lin Zhao, Qingshan Chen

**Affiliations:** 1 State Key Laboratory of Smart Farm Technologies and Systems, Northeast Agricultural University, Harbin 150030, China; zhenyu_xu2002@outlook.com (Z.X.); tangyuecheng0116@163.com (Y.T.); jonnjoe210@gmail.com (H.R.); 2 State Key Laboratory of Black Soils Conservation and Utilization, Key Laboratory of Soybean Molecular Design Breeding, Northeast Institute of Geography and Agroecology, Chinese Academy of Sciences, Harbin 150081, China

**Keywords:** soybean, *GmHIR1*, *Phytophthora sojae*, disease resistance, transcriptome, proteome

## Abstract

*Phytophthora* root rot is a major disease that reduces soybean yield and quality. The role of hypersensitive-induced response (HIR) proteins in soybean resistance to *Phytophthora sojae* (*P. sojae*) remains unclear. In this study, wild-type W82, *GmHIR1* overexpression lines (OE1 and OE2), and CRISPR/Cas9 knockout mutants (Q48 and Q54) were used to characterize *GmHIR1* function. *GmHIR1* was induced after *P. sojae* infection. The knockout mutants showed larger lesions, higher pathogen biomass and increased cell death at infection sites. In contrast, the overexpression lines exhibited reduced lesion size, lower pathogen accumulation, and weaker staining signals. These results indicate that *GmHIR1* positively regulates soybean resistance to *P. sojae*. Further transcriptomic and proteomic analyses showed that *GmHIR1* overexpression caused broad changes in infection-induced molecular responses. Differentially regulated genes and proteins were mainly associated with Ca^2+^ and MAPK signaling, oxidative stress response, peroxidase activity, and phenylpropanoid and flavonoid metabolism. In summary, *GmHIR1* acts as a positive regulator of soybean resistance to *P. sojae*. It may enhance resistance through association with early immune responses, redox homeostasis, programmed cell death regulation, and defense-related metabolic processes. This study identifies *GmHIR1* as a candidate gene for improving soybean resistance to *Phytophthora* root rot.

## 1. Introduction

Soybean is one of the most important oil and protein crops worldwide. Its seeds contain up to 40% protein, making soybean a major source of plant-derived dietary protein [1,2]. However, soybean production is challenged by various biotic stresses, including fungal, bacterial, viral, and oomycete pathogens. These diseases often lead to severe yield losses and represent a major constraint on soybean production [3]. *Phytophthora* root rot, caused by the soil-borne oomycete *Phytophthora sojae* (*P. sojae*), is one of the most destructive soybean diseases [4].

Recent studies have provided increasing insights into the molecular mechanisms underlying the interaction between *P. sojae* and soybean. Like other oomycete pathogens, *P. sojae* secretes a series of effector proteins into host cells to manipulate host immune responses and facilitate infection. Several *P. sojae* effectors have been shown to promote pathogen virulence by targeting key host regulatory processes. For example, PsAvh52 targets the soybean acetyltransferase GmTAP1 and induces its translocation from the cytoplasm to the nucleus. Nuclear-localized GmTAP1 promotes histone H2A and H3 acetylation, thereby activating susceptibility-related gene expression and enhancing *P. sojae* infection [5]. Similarly, PsAvh23 competitively interacts with the soybean histone acetyltransferase subunit ADA2, disrupting the assembly of the ADA2/GCN5 complex. This interference reduces H3K9 acetylation and alters the regulation of host defense genes, ultimately promoting pathogen colonization [6]. In addition, the effector Avr1d suppresses the activity of the soybean E3 ligase GmPUB13 by competing with E2 ubiquitin-conjugating enzymes for GmPUB13 binding, resulting in the stabilization of GmPUB13 and enhanced pathogen infection [7]. These findings highlight the sophisticated strategies employed by *P. sojae* effectors to reprogram host cellular processes and establish infection.

*P. sojae* can infect soybean plants throughout the entire growth period. Seedling infection causes seed decay and damping-off, while infection at later stages leads to root rot, stem browning, leaf chlorosis, wilting or plant death. *P. sojae* exhibits high pathotype diversity and can persist in soil for long periods. Once established in fields, it is difficult to manage and can persist for long periods, posing a serious threat to sustainable soybean production [8]. Therefore, breeding resistant cultivars is one of the most economical and effective strategies for disease control which relies on the identification and utilization of resistance genes.

Plant immunity consists of two interconnected layers: pattern-triggered immunity (PTI) and effector-triggered immunity (ETI) [9]. PTI is the first line of defense and is activated by pattern recognition receptors (PRRs) located at the plasma membrane. These receptors recognize conserved pathogen-associated molecular patterns (PAMPs), such as bacterial flagellin and fungal chitin, and rapidly induce immune responses including reactive oxygen species (ROS) bursts, MAPK cascade activation, and defense gene expression [10,11]. However, many pathogens secrete effector proteins that suppress PTI and facilitate infection. ETI is activated when these effectors are recognized by intracellular resistance (R) proteins [12]. ETI is mediated by nucleotide-binding leucine-rich repeat (NLR) receptors and often triggers a hypersensitive response (HR), a localized programmed cell death that restricts pathogen spread [13].

During HR, plant cells produce large amounts of ROS, activate calcium signaling, and accumulate defense-related metabolites, while strongly inducing immune-related gene expression. HR can also trigger systemic acquired resistance (SAR), thereby enhancing whole-plant disease resistance [14,15]. Hypersensitive-induced response (HIR) proteins are membrane-associated proteins that are strongly induced during the hypersensitive response. They belong to a plant-specific subfamily of the SPFH protein superfamily and are widely conserved in *Arabidopsis*, rice, tobacco, wheat, and other plant species. HIR proteins play important roles in plant growth, development, and immunity [16]. They are mainly localized in specific nanodomains of the plasma membrane and can form homo- and hetero-oligomers. This oligomerization is essential for their biological function [17,18]. Recent studies showed that *Arabidopsis* HIR2 forms stable high-molecular-weight ring-like complexes, supporting its role as a scaffold protein that organizes membrane microdomains and recruits signaling components [19].

HIR proteins are key regulators of plant immune responses. AtHIR1 and AtHIR2 physically interact with the resistance protein RESISTANCE TO PSEUDOMONAS SYRINGAE 2 (RPS2) and are essential components of RPS2-mediated ETI [20]. AtHIR2 is also involved in PTI signaling through interactions with PRRs and co-receptors such as FLAGELLIN-SENSING 2 (FLS2) and BRI1-ASSOCIATED KINASE 1 (BAK1), thereby regulating flg22-induced ROS production [19]. Loss-of-function mutants of *hir2* and *hir4* show reduced ROS bursts induced by flg22 and chitin, resulting in increased susceptibility to *Pseudomonas syringae* pv. tomato DC3000 and *Sclerotinia sclerotiorum*. The *S. sclerotiorum* effector SsPEIE1 targets HIR proteins by competitively binding AtHIR4 and inhibiting its oligomerization, thereby blocking downstream immune signaling and promoting infection [21]. In tobacco, NbHIR2 is a target of the *S. sclerotiorum* xylanase SsXyl2. NbHIR2 promotes the localization of SsXyl2 to the plasma membrane and induces cell death, which facilitates pathogen colonization [22]. These findings suggest that targeting HIR proteins is an important strategy used by pathogens to overcome plant immunity. Further studies in cotton have shown that GbHIR4 interacts with the mannose-binding lectin receptor-like protein GbMBL1.1A, promoting HR-associated programmed cell death and enhancing resistance to *Verticillium dahlia* [23].

Beyond immunity, HIR proteins also participate in hormone signaling networks and plant development. In apple, MdHIR4 interacts with the jasmonate signaling repressor JASMONATE ZIM-DOMAIN 2 (MdJAZ2) and suppresses anthocyanin accumulation by stabilizing JAZ proteins [24]. The immune regulatory function of HIR proteins is also closely associated with salicylic acid (SA) signaling. Silencing of *NbHIR3s* in tobacco downregulates enhanced disease susceptibility 1 (*EDS1*), nonexpressor of PR genes 1 (*NPR1*), and pathogenesis-related protein 1 (*PR1*) expression and reduces SA accumulation, leading to increased susceptibility to rice stripe virus (RSV). In contrast, overexpression of *NbHIR3s* or rice *OsHIR3* activates SA signaling and enhances resistance to viral and bacterial pathogens [25]. The *Arabidopsis hir2* mutant shows reduced hypocotyl length, smaller rosette size, and impaired inflorescence development, whereas *HIR2* overexpression promotes growth, suggesting that HIR2 may regulate plant morphogenesis by modulating membrane dynamics and signaling of growth-related receptors [19].

Research on the soybean *HIR* gene family remains limited. Only a few members have been reported to be induced upon *P. sojae* infection [26]. *GmHIR1* also shows tissue-specific expression patterns at the promoter level [27]. However, the molecular mechanisms underlying their roles in disease resistance remain unclear. To elucidate the function of *GmHIR1* (Glyma.05G029800) in resistance to *P. sojae*, this study used wild-type W82, overexpression lines OE1 and OE2, and *GmHIR1* knockout mutants Q48 and Q54. The expression pattern and infection response of *GmHIR1* were analyzed. Root inoculation assays were performed to evaluate disease resistance. In addition, trypan blue staining, transcriptomic, and proteomic analyses were conducted to characterize defense-related changes in *GmHIR1* overexpression lines. This study provides insights into the role of GmHIR1 proteins in regulating soybean resistance to *P. sojae* and identifies potential targets for molecular breeding of disease-resistant soybean varieties.

## 2. Results

### 2.1. Expression Pattern Analysis of GmHIR1 and Generation of GmHIR1 Transgenic Soybean Plants

**Figure 1:**
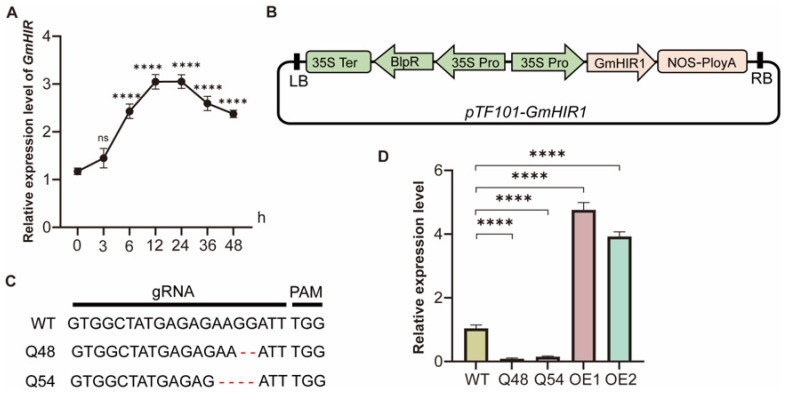
Expression analysis of *GmHIR1* and molecular characterization of knockout and overexpression soybean lines. (**A**) The temporal expression pattern of *GmHIR1* in soybean roots following *P. sojae* inoculation, as determined by RT-qPCR. Statistical significance was determined using one-way ANOVA followed by Dunnett’s multiple comparisons test (ns, not significant; **** *p* < 0.0001) (**B**) A schematic diagram of the *GmHIR1* overexpression vector pTF101-GmHIR1. LB and RB indicate the left and right borders of the T-DNA region, respectively. BlpR denotes the blasticidin resistance gene used as a selectable marker and is independent of the *GmHIR1* expression cassette. (**C**) The sequence alignment of CRISPR/Cas9 target sites in wild-type (WT) and mutant lines. Q48 and Q54 represent independent knockout alleles. Red dashes indicate 2 bp and 4 bp deletions at the target sites. (**D**) Relative expression levels of *GmHIR1* in WT, CRISPR/Cas9 knockout lines (Q48 and Q54), and overexpression lines (OE1 and OE2). Data are presented as mean ± SEM (n = 3 independent biological replicates), and error bars indicate SEM. Statistical significance was determined using one-way ANOVA followed by Tukey’s multiple comparison test. Asterisks indicate significant differences (**** *p* < 0.0001).

To characterize the expression pattern of *GmHIR1* during *P. sojae* infection, RT-qPCR was performed to examine its temporal expression after inoculation. The results showed that *GmHIR1* was gradually upregulated upon infection. Its expression increased significantly at 6 h post-inoculation and remained at a relatively high level from 12 h to 24 h. A slight decrease was observed from 36 to 48 h, although expression still remained higher than the pre-inoculation level (Figure 1A). These results indicate that *GmHIR1* is responsive to *P. sojae* infection and may participate in defense responses.

Since *GmHIR1* may play an important role in *P. sojae* resistance, a *GmHIR1* overexpression construct driven by 35S promoter (pTF101-GmHIR1) was generated. Two independent transgenic lines, OE1 and OE2, were obtained (Figure 1B). In parallel, two independent knockout mutant lines, Q48 and Q54, were generated using the CRISPR/Cas9 system. Sequencing of the target sites confirmed a 2 bp deletion in Q48 and a 4 bp deletion in Q54, resulting in frameshift mutations (Figure 1C). Subsequent RT-qPCR analysis showed that *GmHIR1* transcript levels were significantly reduced in Q48 and Q54 compared with the wild type (WT), whereas OE1 and OE2 exhibited markedly increased expression levels (Figure 1D).

### 2.2. GmHIR1 Positively Regulates Soybean Resistance to P. sojae

**Figure 2:**
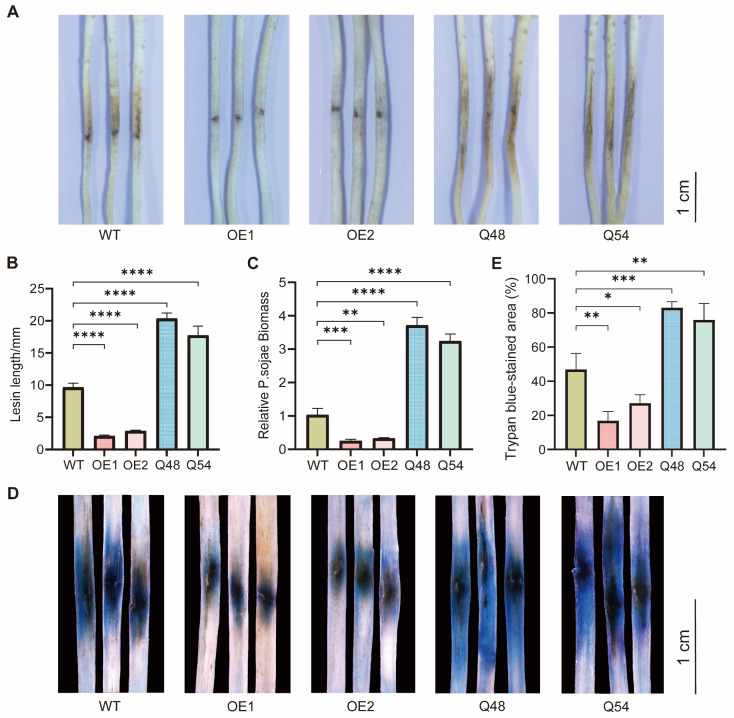
Phenotypic analysis of *GmHIR1* knockout and overexpression lines after *P. sojae* inoculation. (**A**) The disease symptoms of wild-type (WT) lines, overexpression lines (OE1 and OE2), and knockout lines (Q48 and Q54) at 48 h after *P. sojae* inoculation. Scale bars = 1 cm. (**B**) Statistical analysis of lesion lengths in the indicated lines after inoculation. (**C**) Relative biomass of *P. sojae* in the indicated lines, determined by RT-qPCR. Pathogen biomass was normalized using *PsActin* and *GmCYP2* as reference genes. (**D**) Trypan blue staining of infected tissues after *P. sojae* inoculation, showing cell death at infection sites. (**E**) Quantification of trypan blue-stained areas in WT, OE1, OE2, Q48, and Q54 roots at 48 h after *P. sojae* inoculation. The stained area was quantified using ImageJ and expressed as the percentage of the total root area. Data are presented as mean ± SEM (n = 3 independent biological replicates), and error bars indicate SEM. Statistical significance was determined using one-way ANOVA followed by Tukey’s multiple comparison test. Asterisks indicate significant differences (* *p* < 0.05; ** *p* < 0.01; *** *p* < 0.001; **** *p* < 0.0001).

To investigate the role of *GmHIR1* in resistance to *P. sojae*, infection assays were performed using W82, *GmHIR1* overexpression lines and *GmHIR1* knockout mutants. At 48 h post-inoculation, clear phenotypic differences were observed among the genotypes. Compared with WT, both overexpression lines (OE1 and OE2) showed significantly smaller lesion areas, whereas the knockout lines (Q48 and Q54) exhibited markedly larger lesions (Figure 2A). Quantification of lesion length showed a pattern consistent with the visual observations. Lesion lengths in OE1 and OE2 were significantly shorter than those in WT, while *Gmhir1* mutant Q48 and Q54 displayed significantly longer lesion length (Figure 2B). To further assess pathogen growth, relative *P. sojae* biomass was measured by RT-qPCR. The results showed a significant reduction in pathogen accumulation in the overexpression lines, whereas knockout mutants showed increased pathogen biomass compared with WT (Figure 2C). Trypan blue staining revealed differences in infection-associated cell death among the genotypes. Compared with WT, Q48 and Q54 showed more extensive staining at infection sites, whereas OE1 and OE2 exhibited weaker staining signals (Figure 2D). Quantification of the trypan blue-stained area using ImageJ (version 1.54p; National Institutes of Health, USA) further confirmed that the proportion of stained area relative to total root area was significantly increased in *GmHIR1* knockout mutants and decreased in overexpression lines compared with WT (Figure 2E). These results indicate that loss of *GmHIR1* leads to enhanced accumulation of infection-associated cell death, whereas *GmHIR1* overexpression restricts excessive cell death during *P. sojae* infection. Collectively, these findings demonstrate that *GmHIR1* functions as a positive regulator of soybean resistance to *P. sojae*.

### 2.3. GmHIR1 Overexpression Significantly Alters the Transcriptional Response of Soybean to P. sojae Infection

Because the *GmHIR1* knockout mutants showed severe root decay after infection and did not meet the quality requirements for transcriptomic and proteomic analyses, OE1 was selected as the representative overexpression line for subsequent omics analyses due to its higher expression level (Figure 1D) and stable resistant phenotype (Figure 2A). To investigate the transcriptional changes associated with *GmHIR1* overexpression during *P. sojae* infection, W82 and the *GmHIR1* overexpression line OE1 were used for RNA-seq analysis at the V1 stage. Soybean roots were inoculated with *P. sojae* and were collected at 0 h and 48 h post-inoculation (hpi). Root segments of approximately 1 cm centered on the infection site were harvested, with three biological replicates per treatment.

Principal component analysis (PCA) showed clear clustering among biological replicates and distinct separation between genotypes and treatments, indicating high data reproducibility and strong differences among groups (Figure S1A). Detailed RNA-seq quality-control and mapping statistics are provided in Supplemental Table S1. Using non-inoculated samples as control, 2193 differentially expressed genes (DEGs) were identified in W82 after 48 hpi, including 1535 upregulated and 658 downregulated genes (Figure S1B). In OE1, 7824 DEGs were identified under the same conditions, including 4086 upregulated and 3738 downregulated genes (Figure S1C). The complete list of identified DEGs is provided in Supplemental Table S2. Differential expression analysis identified DEGs between W82 and OE1 at 48 hpi, including 2213 upregulated and 2721 downregulated genes (Figure S1D). These results indicate that both genotypes respond transcriptionally to *P. sojae* infection, while OE1 exhibits a substantially larger number of DEGs, suggesting that *GmHIR1* overexpression markedly reshapes the transcriptional response to infection.

**Figure 3:**
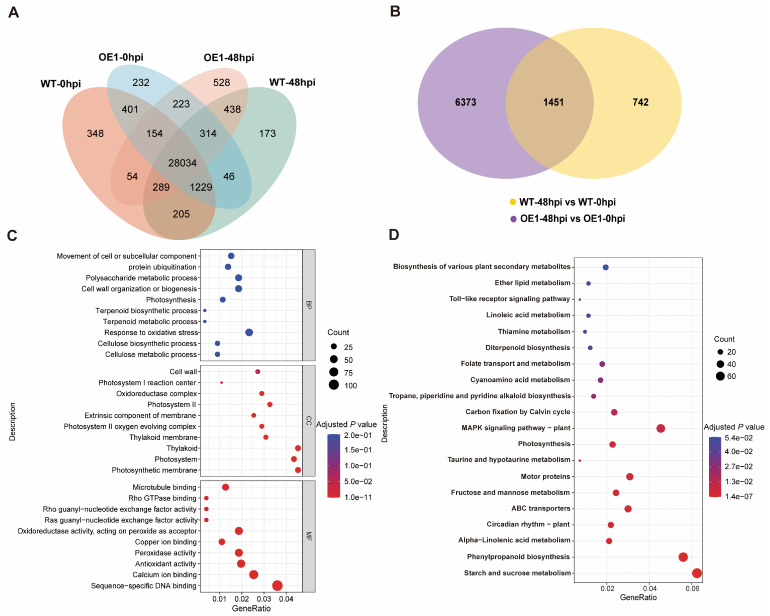
GO and KEGG enrichment analyses of OE1-specific DEGs after *P. sojae* inoculation. (**A**) A Venn diagram showing the overlap of differentially expressed genes (DEGs) identified in WT and OE1 at 0 hpi and 48 hpi after *P. sojae* inoculation. (**B**) A Venn diagram showing shared and OE1-specific DEGs identified in OE1-48hpi vs. OE1-0hpi and WT-48hpi vs. WT-0hpi. OE1-specific DEGs were used for subsequent enrichment analyses. (**C**) Gene Ontology (GO) enrichment analysis of OE1-specific DEGs. The dot size represents the number of genes in each GO term, and the dot color indicates the adjusted *p* value (padj). (**D**) Kyoto Encyclopedia of Genes and Genomes (KEGG) pathway enrichment analysis of OE1-specific DEGs. The dot size represents the number of genes in each pathway, and the dot color indicates the adjusted *p* value (padj).

Comparison of DEGs between W82 and OE1 under control and infection conditions showed both shared and genotype-specific transcriptional responses (Figure 3A). To further explore *GmHIR1* overexpression-specific responses, genes uniquely differentially expressed in OE1 were extracted for Gene Ontology (GO) and Kyoto Encyclopedia of Genes and Genomes (KEGG) enrichment analyses (Figure 3B). GO enrichment analysis showed that these *GmHIR1* overexpression-specific DEGs were mainly associated with molecular functions including sequence-specific DNA binding, calcium ion binding, copper ion binding, peroxidase activity, antioxidant activity, oxidoreductase activity acting on peroxide as acceptor, and microtubule binding. In terms of cellular components, they were mainly enriched in photosystem, photosynthetic membrane, and oxidoreductase complex (Figure 3C). The complete GO enrichment results are provided in Supplemental Table S3. KEGG pathway analysis further revealed significant enrichment in starch and sucrose metabolism, phenylpropanoid biosynthesis, the MAPK signaling pathway, ABC transporters, fructose and mannose metabolism, and photosynthesis (Figure 3D). The complete KEGG enrichment results are provided in Supplemental Table S4. Collectively, these results indicate that *GmHIR1* overexpression is associated with extensive transcriptional changes during *P. sojae* infection.

### 2.4. GmHIR1 Overexpression Alters the Proteomic Response of Soybean to P. sojae Infection

**Figure 4:**
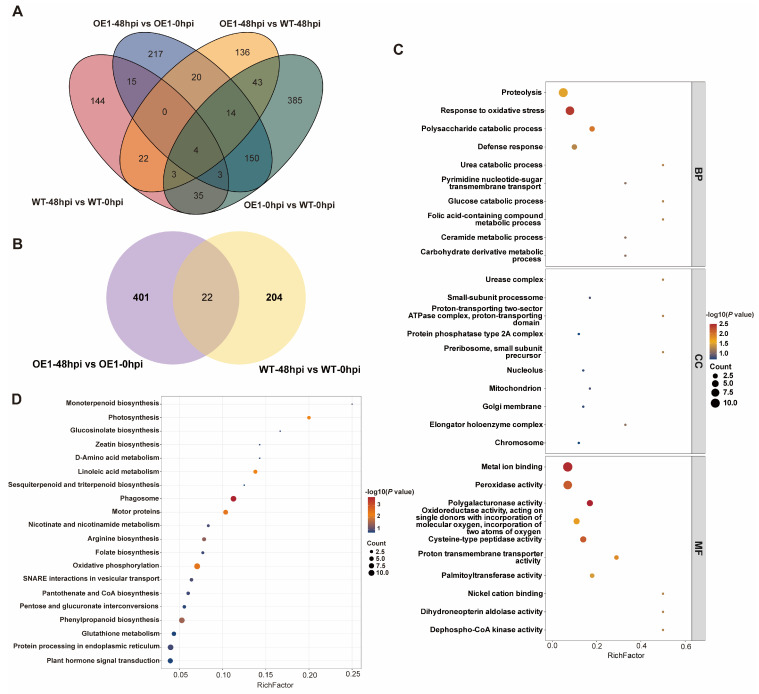
Comparative proteomic analysis of OE1 and W82 after *P. sojae* inoculation. (**A**) A Venn diagram showing the overlap of differentially expressed proteins (DEPs) identified in four pairwise comparisons. (**B**) A Venn diagram showing shared and genotype-specific DEPs identified in OE1 and W82 in response to *P. sojae* inoculation at 48 hpi. (**C**) Gene Ontology (GO) enrichment analysis of OE1-specific DEPs, grouped into biological process (BP), cellular component (CC), and molecular function (MF) categories. The dot size indicates the number of proteins, and the dot color represents enrichment significance [−log10(*p* value)]. (**D**) Kyoto Encyclopedia of Genes and Genomes (KEGG) pathway enrichment analysis of OE1-specific DEPs. The dot size indicates the number of proteins, and the dot color represents enrichment significance [−log10(*p* value)].

To investigate protein-level changes associated with *GmHIR1* overexpression, proteomic profiling of OE1 and W82 was conducted under control conditions and following 48 h of *P. sojae* infection. Principal component analysis (PCA) showed clear separation among genotypes and treatments, and biological replicates clustered tightly, indicating good reproducibility and reliability of the proteomic data (Figure S2A). Additional proteomic quality-control information is provided in Supplemental Table S5. Differential protein analysis revealed pronounced changes in protein abundance in both OE1 and W82 after infection. Notably, OE1 exhibited a greater number of differentially expressed proteins than W82 (Figure S2B). Venn analysis was further used to identify OE1-specific differentially expressed proteins (Figure 4A,B). The complete DEP datasets are provided in Supplemental Table S6. A total of 22 proteins were commonly regulated in both genotypes upon infection. In contrast, 401 proteins were uniquely regulated in OE1, whereas 204 proteins were specific to W82. These results indicate distinct proteomic responses to *P. sojae* infection between the two genotypes.

To explore the potential functions of OE1-specific differentially expressed proteins, GO and KEGG enrichment analyses were performed (Figure 4C,D). GO enrichment analysis showed that these proteins were mainly associated with biological processes such as response to oxidative stress and the polysaccharide catabolic process. In terms of molecular function, they were enriched in metal ion binding, peroxidase activity, polygalacturonase activity, and cysteine-type peptidase activity (Figure 4C). The complete GO enrichment results for DEPs are provided in Supplemental Table S7. KEGG pathway analysis further indicated that these proteins were mainly enriched in phagosome, oxidative phosphorylation, motor proteins, linoleic acid metabolism, photosynthesis, and phenylpropanoid biosynthesis pathways (Figure 4D). The complete KEGG enrichment results for DEPs are provided in Supplemental Table S8. Together, these results suggest that overexpression of *GmHIR1* reshapes the proteomic response of soybean during *P. sojae* infection.

### 2.5. Integrated Transcriptomic and Proteomic Analyses Reveal Coordinated Changes in Defense Metabolism in GmHIR1 Overexpression Lines

**Figure 5:**
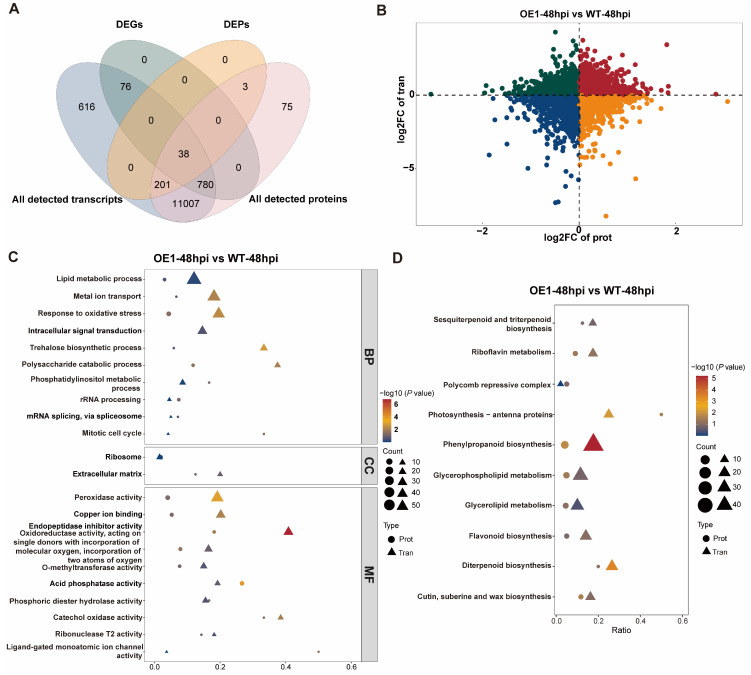
Integrated transcriptomic and proteomic analysis of OE1 and W82(WT) at 48 hpi. (**A**) Venn diagram showing the overlap among all detected transcripts, differentially expressed genes (DEGs), all detected proteins, and differentially expressed proteins (DEPs) between OE1 and WT at 48 hpi. All detected transcripts represent genes identified by RNA-seq; DEGs represent differentially expressed genes; all detected proteins represent proteins identified by proteomic analysis; and DEPs represent differentially expressed proteins. (**B**) Nine-quadrant plot showing the relationship between transcript and protein abundance changes in OE1 48 hpi vs WT 48 hpi. Colors indicate different regulatory patterns between transcript and protein levels. Red and blue represent concordant upregulation and downregulation, respectively, whereas green and orange represent discordant regulation between transcript and protein levels. (**C**) GO enrichment analysis of molecules identified in the integrated transcriptomic and proteomic analysis. The circles represent proteins and the triangles represent transcripts. The dot color indicates enrichment significance [−log10(*p* value)], and the dot size represents the number of genes or proteins. (**D**) KEGG pathway enrichment analysis of molecules identified in the integrated analysis. The circles represent proteins and the triangles represent transcripts. The dot color indicates enrichment significance [−log10(*p* value)], and the dot size represents the number of genes or proteins.

To further investigate coordinated changes at both transcriptional and protein levels in *GmHIR1* overexpression lines, an integrated analysis of transcriptomic and proteomic data was performed using OE1 and W82 samples collected at 48 h post-inoculation with *P. sojae*. A total of 38 molecules were identified as differentially regulated at both mRNA and protein levels (Figure 5A). The detailed information of these 38 co-differential transcript/protein molecules is provided in Supplemental Table S9. Nine-quadrant analysis showed that a subset of molecules displayed consistent trends between transcript and protein abundance, whereas others showed discordant changes. This indicates that the differences between OE1 and W82 are not only regulated at the transcriptional level, but may also involve post-transcriptional and post-translational regulation (Figure 5B).

GO enrichment analysis revealed that these co-differential molecules were mainly associated with biological processes including response to oxidative stress, metal ion transport, the trehalose biosynthetic process, the polysaccharide catabolic process, and the lipid metabolic process. In terms of molecular function, they were enriched in endopeptidase inhibitor activity, peroxidase activity, copper ion binding, and acid phosphatase activity (Figure 5C). KEGG pathway analysis further indicated enrichment in phenylpropanoid biosynthesis, photosynthesis–antenna proteins, diterpenoid biosynthesis, and lipid metabolism-related pathways, as well as flavonoid biosynthesis (Figure 5D). Collectively, these results suggest that *GmHIR1* overexpression was associated with changes in defense-related secondary metabolism and oxidative stress responses.

## 3. Discussion

### 3.1. The Role of GmHIR1 in Soybean Resistance to P. sojae

This study demonstrates that *GmHIR1* functions as a positive regulator of soybean resistance to *P. sojae*. *GmHIR1* showed clear inducible expression after pathogen inoculation, suggesting its involvement in the soybean response to infection. Consistent with this expression pattern, phenotypic analyses revealed that the overexpression lines OE1 and OE2 displayed significantly reduced lesion size and decreased pathogen biomass, whereas the CRISPR/Cas9 knockout lines Q48 and Q54 showed enlarged lesions and increased pathogen accumulation. These results indicate that loss of *GmHIR1* enhances disease susceptibility, while its overexpression improves resistance.

This finding is consistent with previous reports on *HIR* genes in other plant species. *HIR* family members have been shown to function as positive regulators of plant immunity, where loss-of-function or silencing generally compromises resistance, whereas increased expression enhances defense against fungal, bacterial, or viral pathogens [21,23,25]. For example, in wheat, *TaHIR1* and *TaHIR3* are upregulated upon stripe rust infection and contribute to resistance by promoting hypersensitive response and defense gene activation. Silencing of these genes using BSMV-VIGS significantly reduces resistance [28]. In maize, *ZmHIR3* contributes to resistance against Gibberella stalk rot. Its loss-of-function mutant shows increased susceptibility [29]. Co-expression network analysis further links this gene to multiple defense-related genes [29]. In rapeseed, heterologous expression of *BnHIR2.7* in *Arabidopsis* enhances resistance to *Sclerotinia sclerotiorum* [30]. Together, these studies support a conserved positive role of HIR genes in plant immunity.

### 3.2. GmHIR1 Is Involved in Hypersensitive Response and Programmed Cell Death at Infection Sites

The hypersensitive response (HR) is a form of localized programmed cell death (PCD) that restricts pathogen spread by limiting infection to a small number of cells. However, the effectiveness of HR depends on its spatial confinement. Excessive cell death may reflect tissue damage rather than enhanced resistance. Therefore, trypan blue staining should be interpreted together with lesion size and pathogen biomass [31]. In this study, the *GmHIR1* knockout mutant Q48 exhibited stronger and more extensive trypan blue staining after infection, together with larger lesions and higher pathogen biomass. These results suggest that the increased cell death in the mutant does not effectively restrict pathogen growth. Instead, it likely reflects aggravated tissue damage and dysregulated PCD. In contrast, *GmHIR1* overexpression lines showed weaker staining and reduced disease symptoms, indicating that GmHIR1 helps maintain a more controlled and localized cell death response.

Previous studies also support a role for HIR proteins in HR-PCD regulation. In rice, OsHIR1 localizes to the plasma membrane and its ectopic expression induces hypersensitive cell death and enhances resistance [32]. In pepper, CaHIR1 is also involved in immune-related cell death regulation [33]. Moreover, viral effector proteins can suppress HR by targeting HIR proteins. For example, the C4 protein of begomovirus interferes with HIR1 self-association and promotes its degradation, thereby suppressing HR [34]. These findings indicate that HIR-mediated cell death is a key component of plant immunity and a common target of pathogen effectors. This interpretation is consistent with the infection strategy of *P. sojae*, a hemibiotrophic pathogen. The timing and extent of host cell death strongly influence pathogen colonization and expansion. *P. sojae* effectors have been shown to modulate host cell death processes, highlighting PCD as a central process for soybean and oomycete interactions [35]. Therefore, the enhanced cell death observed in the knockout mutant, together with increased lesion size and pathogen biomass, suggests that uncontrolled PCD may facilitate disease progression. Overall, GmHIR1 likely contributes to soybean resistance by maintaining the spatial restriction and appropriate level of HR-associated PCD.

### 3.3. GmHIR1 Overexpression Is Associated with Changes in Ca^2+^/MAPK-Related Defense Responses

Ca^2+^ signaling and MAPK cascades are among the earliest immune signaling events following pathogen perception. They transmit signals from the plasma membrane to the nucleus and regulate defense gene expression [36,37]. In this study, OE1 exhibited a markedly higher number of differentially expressed genes compared with W82 after infection, indicating that *GmHIR1* overexpression amplifies transcriptional responses to *P. sojae*.

GO enrichment analysis further showed that OE1-specific DEGs were significantly associated with calcium ion binding, the MAPK signaling pathway, and sequence-specific DNA binding. Collectively, these results suggest that Ca^2+^/MAPK-related genes and pathways are associated with the altered defense responses observed in OE1 during infection.

This is consistent with previous studies. For example, overexpression of GmWAK1 enhances resistance to *P. sojae* by promoting Ca^2+^ accumulation and linking to GmMPK6-associated signaling through its interacting protein GmANNRJ4 [38]. In addition, the GmMKK4-GmMPK6-GmERF113 module has been shown to enhance resistance by stabilizing and activating defense transcription factors that induce PR genes such as *GmPR1* and *GmPR10-1* [39]. Together with the enhanced resistance phenotype observed in OE1, these results suggest that enhanced Ca^2+^ signaling and MAPK cascade activation may strengthen defense transcriptional responses during infection.

### 3.4. Overexpression of GmHIR1 May Enhance Defense-Related Secondary Metabolism

Defense-related secondary metabolism is a key component of plant immunity. Phenylpropanoid, flavonoid, and isoflavonoid pathways contribute to the biosynthesis of lignin, flavonoids, and soybean phytoalexins such as glyceollins. In this study, integrated transcriptomic and proteomic analyses revealed enrichment in phenylpropanoid biosynthesis, diterpenoid biosynthesis, and flavonoid biosynthesis. These results suggest that *GmHIR1* overexpression may enhance defense-related secondary metabolic reprogramming and may also influence metabolite transport during infection. Previous studies support the importance of these pathways in resistance to *P. sojae*. *GmDIR22* promotes lignan biosynthesis through the phenylpropanoid pathway and enhances resistance [40]. GmCHI1A, a key enzyme in flavonoid biosynthesis, improves resistance when overexpressed [41]. Collectively, these studies highlight phenylpropanoid-derived metabolites as central components of soybean defense. In summary, *GmHIR1* overexpression is associated with changes in phenylpropanoid, flavonoid, and isoflavonoid metabolic pathways, which may contribute to enhanced soybean resistance during infection.

## 4. Materials and Methods

### 4.1. Soybean Transformation

The *GmHIR1* knockout mutants were generated in the Williams 82 background using the CRISPR/Cas9 system. The target region was amplified by PCR and sequenced to identify mutations. Two independent homozygous mutant lines, Q48 and Q54, carrying frameshift mutations caused by 2 bp and 4 bp deletions, respectively, were selected for subsequent experiments. The homozygous genotype of each mutant line was confirmed by sequencing of the target region.

For the generation of *GmHIR1* overexpression lines, the full-length coding sequence of *GmHIR1* was cloned into the pTF101 expression vector and introduced into soybean plants. Independent transgenic lines were identified by PCR amplification of the transgene and confirmed by RT-qPCR analysis of GmHIR1 expression. Two independent overexpression lines, OE1 and OE2, with increased *GmHIR1* expression levels were selected for functional analysis.

All the primers are listed in Supplemental Table S10. Briefly, the pTF101-*GmHIR1* construct and CRISPR-*GmHIR1* were introduced into *Agrobacterium tumefaciens* strain EHA101. Transgenic soybean plants harboring the construct were regenerated following previously described protocols [42].

### 4.2. Plant Materials and Growth Conditions

Soybean (*Glycine max*) cultivar Williams 82 (W82) was used as the wild-type control in this study. Soybean seeds of similar size were sown in a sterilized mixture of vermiculite and soil (1:1, *v*/*v*). The plants were grown in a controlled-environment chamber under a 16 h light/8 h dark photoperiod, with a day/night temperature of 25 °C/22 °C and relative humidity of 60–70%. The seedlings were grown to the V1 stage, at which the first trifoliate leaf was fully expanded, before pathogen inoculation or sample collection.

### 4.3. P. sojae Inoculation and Disease Resistance Assay

A *Phytophthora sojae* isolate maintained in our laboratory was used for inoculation in this study. The isolate was maintained on V8 agar medium at 25 °C in the dark for 5–7 days.

Soybean seeds were germinated in darkness for 4 days to obtain etiolated seedlings for inoculation. A wound was made on the hypocotyl approximately 3 cm below the stem base using a sterile 1 mL syringe needle. Agar plugs (7.5 mm in diameter) were excised from the actively growing margin of the *P. sojae* colony and placed directly onto the wounded site. Each seedling received one agar plug of identical size prepared from colonies cultured under the same conditions to ensure inoculum standardization. After inoculation, the seedlings were covered with moist gauze and plastic film to maintain high humidity and incubated in the dark at 28 °C under approximately 90% relative humidity for 48 h.

Disease symptoms were photographed at 48 h post-inoculation (hpi). Lesion length was measured as the maximum distance between the two outermost visible boundaries of the necrotic lesion. Relative *P. sojae* biomass was quantified by quantitative PCR using the pathogen-specific marker gene *PsACT* and normalized to the soybean reference gene *GmCYP2*. Relative pathogen biomass was calculated using the 2^−ΔΔCt^ method.

### 4.4. Gene Expression Analysis by RT-qPCR

Total RNA was extracted from soybean roots using the FastPure Universal Plant Total RNA Isolation Kit (Vazyme Biotech Co., Ltd., Nanjing, China) according to the manufacturer’s instructions. First-strand cDNA was synthesized using a commercial reverse transcription kit. The resulting cDNA was used as the template for quantitative real-time PCR (qRT-PCR) with a SYBR Green-based detection system. *GmActin* was used as the internal reference gene, and relative expression levels were calculated using the 2^−ΔΔCt^ method. Three independent biological replicates were performed for each treatment, and each biological replicate consisted of pooled samples collected from independently grown plants.

### 4.5. Trypan Blue Staining

The trypan blue staining assay was performed according to previous protocol [43]. Soybean roots were collected at 48 hpi with *P. sojae* and immersed in trypan blue staining solution. The samples were vacuum-infiltrated at −0.08 MPa for 20 min, rinsed with PBS buffer, and observed under a stereomicroscope. Representative images were captured for analysis. For quantitative analysis, trypan blue-stained areas were measured using ImageJ (version 1.54p; National Institutes of Health, USA). The entire root region was manually selected as the region of interest (ROI), and the stained area was extracted using the Color Threshold function based on the HSB color space. The stained regions were identified according to consistent color-selection criteria. The proportion of trypan blue-stained area was calculated as the percentage of stained area relative to the total root area. Three biological replicates were analyzed for each genotype.

### 4.6. RNA-Seq Analysis

RNA-seq analysis was performed using the *GmHIR1*-overexpressing line OE1 and wild-type W82. Plants were grown to the V1 stage under the conditions described above. Root samples were collected at 0 h and 48 h after *P. sojae* inoculation. For 0 h mock controls, hypocotyls were subjected to the same mechanical wounding procedure using a sterile syringe needle but without *P. sojae* inoculation, and samples were collected immediately after mock treatment. For each biological replicate, roots from multiple soybean plants were collected and pooled as one sample. At 48 h post-inoculation, approximately 1 cm root segments surrounding the inoculation sites were harvested. Three independent biological replicates were prepared for each genotype and treatment, with each replicate derived from independently grown plants.

Total RNA was extracted using an RNA extraction kit according to the manufacturer’s instructions. RNA integrity and concentration were assessed using an Agilent 2100 Bioanalyzer (Agilent Technologies, Santa Clara, CA, USA). Only high-quality RNA samples were used for library construction. mRNA was enriched using oligo(dT) magnetic beads, followed by cDNA library construction according to the Illumina protocol [44]. Library quality and insert size were evaluated using an Agilent 2100 Bioanalyzer, and concentrations were determined using Qubit fluorometry and RT-qPCR. Library concentrations were determined using a Qubit 2.0 Fluorometer (Thermo Fisher Scientific, Waltham, MA, USA) and RT-qPCR. Libraries were sequenced on a DNBSEQ-T7 platform (MGI Tech Co., Ltd., Shenzhen, China) to generate 150 bp paired-end reads. Raw reads were filtered to remove adapters, ambiguous reads, and low-quality reads [45]. Clean reads were aligned to the soybean reference genome (*Glycine max* Wm82.a4.v1) using HISAT2 (version 2.2.1) [46], and gene counts were obtained using featureCounts (version 2.0.6) [47]. Differentially expressed genes (DEGs) were identified using DESeq2 (version 1.42.0) [48], with thresholds of |log2(fold change)| ≥ 1 and FDR < 0.05. DEGs were used for downstream GO and KEGG analyses.

### 4.7. GO and KEGG Enrichment Analysis

GO and KEGG enrichment analyses were performed to investigate the biological functions of differentially expressed genes (DEGs), differentially expressed proteins (DEPs), and shared differentially changed molecules identified from integrative transcriptomic and proteomic analysis. GO analysis included biological process, molecular function, and cellular component categories. KEGG analysis was used to identify enriched metabolic and signaling pathways. Enrichment significance was evaluated using a hypergeometric test, and *p* values were adjusted using the Benjamini–Hochberg method. Terms with padj or FDR < 0.05 were considered significantly enriched.

### 4.8. Statistical Analysis

All experiments were performed with at least three independent biological replicates. Data are presented as mean ± standard error (SEM). Statistical significance was determined using one-way ANOVA followed by Dunnett’s multiple comparison test. Differences were considered statistically significant at *p* < 0.05. Analyses were performed using GraphPad Prism (version 10.1.2)and R software (version 4.3.1).

### 4.9. Proteomic Analysis

Proteomic analysis was performed using root samples from OE1 and W82 under control conditions and at 48 h after *P. sojae* inoculation. Each biological replicate consisted of pooled root tissues collected from independently grown plants. Three biological replicates were analyzed for each genotype and treatment. Total proteins were extracted and quantified prior to enzymatic digestion. Peptides were analyzed using a Vanquish Neo LC system coupled with an Orbitrap Astral mass spectrometer (Thermo Fisher Scientific, Bremen, Germany). Mass spectrometry data were processed using DIA-NN software (version 1.8) and searched against the soybean protein database containing 73,840 sequences. Peptide and protein identifications were filtered using a target-decoy strategy, and proteins with a Q.Value greater than 1% were removed to ensure an identification confidence greater than 99%. Protein quantitative values were normalized using maximum peak normalization, and missing values were not imputed. Differentially expressed proteins (DEPs) were identified based on fold change and statistical significance. Proteins with a fold change ≥ 1.5 or ≤0.67 and *p* < 0.05 were considered significantly differentially expressed. No *p*-value adjustment was applied for DEP screening. DEPs were subsequently subjected to GO and KEGG enrichment analyses.

## Data Availability

The original contributions presented in this study are included in the article/Supplementary Material. Further inquiries can be directed to the corresponding author.

## Supplementary Materials

The following supporting information can be downloaded at https://www.mdpi.com/article/10.3390/plants15142211/s1, Figure S1: Transcriptome profiling and identification of differentially expressed genes in W82 and OE1 following *Phytophthora sojae* infection; Figure S2: Global proteomic variation and differentially expressed proteins in OE1 and W82 after *Phytophthora sojae* inoculation; Table S1: RNA-seq quality and mapping statistics; Table S2: RNA-seq differentially expressed genes (DEGs); Table S3: RNA-seq GO enrichment analysis; Table S4: RNA-seq KEGG enrichment analysis; Table S5: Proteomics quality statistics; Table S6: Differentially expressed proteins (DEPs); Table S7: Proteomics GO enrichment analysis; Table S8: Proteomics KEGG enrichment analysis; Table S9: Co-differential molecules between OE1 and W82 at 48 hpi; Table S10: Primer sequences.

## References

[1] Ramlal A., Nautiyal A., Lal S., Chigeza G. (2023). A wonder legume, soybean: Prospects for improvement. Front. Plant Sci..

[2] Rotundo J.L., Marshall R., McCormick R., Truong S.K., Styles D., Gerde J.A., Gonzalez-Escobar E., Carmo-Silva E., Janes-Bassett V., Logue J. (2024). European soybean to benefit people and the environment. Sci. Rep..

[3] Hale B., Brown E., Wijeratne A. (2023). An updated assessment of the soybean–*Phytophthora sojae* pathosystem. Plant Pathol..

[4] Lin F., Li W., McCoy A.G., Gao X., Collins P.J., Zhang N., Wen Z., Cao S., Wani S.H., Gu C. (2021). Molecular mapping of quantitative disease resistance loci for soybean partial resistance to *Phytophthora sansomeana*. Theor. Appl. Genet..

[5] Li H., Wang H., Jing M., Zhu J., Guo B., Wang Y., Lin Y., Chen H., Kong L., Ma Z. (2018). A *Phytophthora* effector recruits a host cytoplasmic transacetylase into nuclear speckles to enhance plant susceptibility. Elife.

[6] Kong L., Qiu X., Kang J., Wang Y., Chen H., Huang J., Qiu M., Zhao Y., Kong G., Ma Z. (2017). A *Phytophthora* effector manipulates host histone acetylation and reprograms defense gene expression to promote infection. Curr. Biol..

[7] Lin Y., Hu Q., Zhou J., Yin W., Yao D., Shao Y., Zhao Y., Guo B., Xia Y., Chen Q. (2021). *Phytophthora sojae* effector Avr1d functions as an E2 competitor and inhibits ubiquitination activity of GmPUB13 to facilitate infection. Proc. Natl. Acad. Sci. USA.

[8] Akamatsu H., Kato M., Ochi S., Mimuro G., Matsuoka J.-i., Takahashi M. (2019). Variation in the resistance of Japanese soybean cultivars to *Phytophthora* root and stem rot during the early plant growth stages and the effects of a fungicide seed treatment. Plant Pathol. J..

[9] Peng Y., van Wersch R., Zhang Y. (2018). Convergent and divergent signaling in PAMP-triggered immunity and effector-triggered immunity. Mol. Plant-Microbe Interact..

[10] Jones J.D., Dangl J.L. (2006). The plant immune system. Nature.

[11] Singla-Rastogi M. (2025). The complex immune puzzle: A deeper dive into the MORC1-mediated broad-spectrum defense signaling pathway. Plant Cell.

[12] Yu X.Q., Niu H.Q., Liu C., Wang H.L., Yin W., Xia X. (2024). PTI-ETI synergistic signal mechanisms in plant immunity. Plant Biotechnol. J..

[13] Cui H., Tsuda K., Parker J.E. (2015). Effector-triggered immunity: From pathogen perception to robust defense. Annu. Rev. Plant Biol..

[14] Fu Z.Q., Dong X. (2013). Systemic acquired resistance: Turning local infection into global defense. Annu. Rev. Plant Biol..

[15] Ngou B.P.M., Ahn H.-K., Ding P., Jones J.D. (2021). Mutual potentiation of plant immunity by cell-surface and intracellular receptors. Nature.

[16] Daněk M., Valentová O., Martinec J. (2016). Flotillins, Erlins, and HIRs: From animal base camp to plant new horizons. Crit. Rev. Plant Sci..

[17] Daněk M., Angelini J., Malínská K., Andrejch J., Amlerová Z., Kocourková D., Brouzdová J., Valentová O., Martinec J., Petrášek J. (2020). Cell wall contributes to the stability of plasma membrane nanodomain organization of *Arabidopsis thaliana* FLOTILLIN2 and HYPERSENSITIVE INDUCED REACTION1 proteins. Plant J..

[18] Daněk M., Hdedeh O., Amo J., Boutet J., Neubergerová M., Safi H., Abuzeineh A., Martín-Barranco A., Fiche J.b., Mercier C. (2026). Mechanisms controlling the plasma membrane targeting and the nanodomain organization of the plant SPFH protein HIR2. Plant J..

[19] Weber H., Ehinger A., Kolb D., Fallahzadeh-Mamaghani V., Halter T., Franz-Wachtel M., zur Oven-Krockhaus S., Gronnier J., Zipfel C., Harter K. (2025). *Arabidopsis* HYPERSENSITIVE INDUCED REACTION 2 affects plasma membrane receptor pathways and organization. bioRxiv.

[20] Qi Y., Tsuda K., Nguyen L.V., Wang X., Lin J., Murphy A.S., Glazebrook J., Thordal-Christensen H., Katagiri F. (2011). Physical association of *Arabidopsis* hypersensitive induced reaction proteins (HIRs) with the immune receptor RPS2. J. Biol. Chem..

[21] Liu X., Zhao H., Yuan M., Li P., Xie J., Fu Y., Li B., Yu X., Chen T., Lin Y. (2024). An effector essential for virulence of necrotrophic fungi targets plant HIRs to inhibit host immunity. Nat. Commun..

[22] Wang P., Wang Y., Hu Y., Chen Z., Han L., Zhu W., Tian B., Fang A., Yang Y., Bi C. (2024). Plant hypersensitive induced reaction protein facilitates cell death induced by secreted xylanase associated with the pathogenicity of *Sclerotinia sclerotiorum*. Plant J..

[23] Yuan L., Wu M., Tan D., Zhang S., Zhang H., Li J., Xia G., Wang F. (2025). Mannose-binding lectin 1. 1A interacts with hypersensitive-induced response 4 to promote hypersensitive cell death and defense responses in cotton upon *Verticillium dahliae* infection. Plant J..

[24] Chen K.-Q., Zhao X.-Y., An X.-H., Tian Y., Liu D.-D., You C.-X., Hao Y.-J. (2017). MdHIR proteins repress anthocyanin accumulation by interacting with the MdJAZ2 protein to inhibit its degradation in apples. Sci. Rep..

[25] Li S., Zhao J., Zhai Y., Yuan Q., Zhang H., Wu X., Lu Y., Peng J., Sun Z., Lin L. (2019). The hypersensitive induced reaction 3 (HIR 3) gene contributes to plant basal resistance via an EDS 1 and salicylic acid-dependent pathway. Plant J..

[26] Xiang Y., Song M., Zhang M., Cao S., Han H. (2015). Molecular characterization of three hypersensitive-induced reaction genes that respond to *Phytophthora sojae* infection in *Glycine max* L. Merr. Legume Res.-Int. J..

[27] Koellhoffer J.P., Xing A., Moon B.P., Li Z. (2015). Tissue-specific expression of a soybean hypersensitive-induced response (HIR) protein gene promoter. Plant Mol. Biol..

[28] Duan Y., Guo J., Shi X., Guan X., Liu F., Bai P., Huang L., Kang Z. (2013). Wheat hypersensitive-induced reaction genes TaHIR1 and TaHIR3 are involved in response to stripe rust fungus infection and abiotic stresses. Plant Cell Rep..

[29] Sun Y., Ruan X., Wang Q., Zhou Y., Wang F., Ma L., Wang Z., Gao X. (2021). Integrated gene co-expression analysis and metabolites profiling highlight the important role of ZmHIR3 in maize resistance to *Gibberella* stalk rot. Front. Plant Sci..

[30] Li M., Tang Y., Yu M., Fan Y., Khan S.U., Chang W., Li X., Wei S., Wei L., Qu C. (2022). Systematic characterization of *Brassica napus* HIR gene family reveals a positive role of BnHIR2. 7 in *Sclerotinia sclerotiorum* resistance. Horticulturae.

[31] Noman A., Aqeel M., Qari S.H., Al Surhanee A.A., Yasin G., Alamri S., Hashem M., Al-Saadi A.M. (2020). Plant hypersensitive response vs pathogen ingression: Death of few gives life to others. Microb. Pathog..

[32] Zhou L., Cheung M.-Y., Li M.-W., Fu Y., Sun Z., Sun S.-M., Lam H.-M. (2010). Rice hypersensitive induced reaction protein 1 (OsHIR1) associates with plasma membrane and triggers hypersensitive cell death. BMC Plant Biol..

[33] Choi H.W., Kim Y.J., Hwang B.K. (2011). The hypersensitive induced reaction and leucine-rich repeat proteins regulate plant cell death associated with disease and plant immunity. Mol. Plant-Microbe Interact..

[34] Mei Y., Ma Z., Wang Y., Zhou X. (2020). Geminivirus C4 antagonizes the HIR1-mediated hypersensitive response by inhibiting the HIR1 self-interaction and promoting degradation of the protein. New Phytol..

[35] Jing M., Guo B., Li H., Yang B., Wang H., Kong G., Zhao Y., Xu H., Wang Y., Ye W. (2016). A *Phytophthora sojae* effector suppresses endoplasmic reticulum stress-mediated immunity by stabilizing plant binding immunoglobulin proteins. Nat. Commun..

[36] Dodds P.N., Chen J., Outram M.A. (2024). Pathogen perception and signaling in plant immunity. Plant Cell.

[37] Wang Q., Cang X., Yan H., Zhang Z., Li W., He J., Zhang M., Lou L., Wang R., Chang M. (2024). Activating plant immunity: The hidden dance of intracellular Ca^2+^ stores. New Phytol..

[38] Zhao M., Li N., Chen S., Wu J., He S., Zhao Y., Wang X., Chen X., Zhang C., Fang X. (2023). GmWAK1, novel wall-associated protein kinase, positively regulates response of soybean to *Phytophthora sojae* infection. Int. J. Mol. Sci..

[39] Gao H., Jiang L., Du B., Ning B., Ding X., Zhang C., Song B., Liu S., Zhao M., Zhao Y. (2022). GmMKK4-activated GmMPK6 stimulates GmERF113 to trigger resistance to *Phytophthora sojae* in soybean. Plant J..

[40] Li N., Zhao M., Liu T., Dong L., Cheng Q., Wu J., Wang L., Chen X., Zhang C., Lu W. (2017). A novel soybean dirigent gene GmDIR22 contributes to promotion of lignan biosynthesis and enhances resistance to *Phytophthora sojae*. Front. Plant Sci..

[41] Zhou Y., Huang J.-L., Zhang X.-L., Zhu L.-M., Wang X.-F., Guo N., Zhao J.-M., Xing H. (2018). Overexpression of chalcone isomerase (CHI) increases resistance against *Phytophthora sojae* in soybean. J. Plant Biol..

[42] Li Y., Fang Q., Cao Y., Yang M., Wang J., Wang M., Li N., Meng F. (2026). Identification and Functional Characterization of Soybean Microexon in Response to Saline-Alkali Stress. Plant Cell Environ..

[43] Fernández-Bautista N., Domínguez-Núñez J.A., Moreno M.M.C., Berrocal-Lobo M. (2016). Plant tissue trypan blue staining during phytopathogen infection. Bio-Protocol.

[44] Parkhomchuk D., Borodina T., Amstislavskiy V., Banaru M., Hallen L., Krobitsch S., Lehrach H., Soldatov A. (2009). Transcriptome analysis by strand-specific sequencing of complementary DNA. Nucleic Acids Res..

[45] Garber M., Grabherr M.G., Guttman M., Trapnell C. (2011). Computational methods for transcriptome annotation and quantification using RNA-seq. Nat. Methods.

[46] Kim D., Langmead B., Salzberg S.L. (2015). HISAT: A fast spliced aligner with low memory requirements. Nat. Methods.

[47] Liao Y., Smyth G.K., Shi W. (2014). featureCounts: An efficient general purpose program for assigning sequence reads to genomic features. Bioinformatics.

[48] Love M.I., Huber W., Anders S. (2014). Moderated estimation of fold change and dispersion for RNA-seq data with DESeq2. Genome Biol..

