# Supplementary material for: Overexpression of GmHIR1 in Soybean Enhances Phytophthora sojae Resistance
Source: Plants (Basel). 2026 Jul 20;15(14):2211. doi: 10.3390/plants15142211 (PMC13416582; doi:10.3390/plants15142211)
Supplement: Supplementary file 1 [file plants-15-02211-s001.zip › Supplementary Figures Revised.pdf]

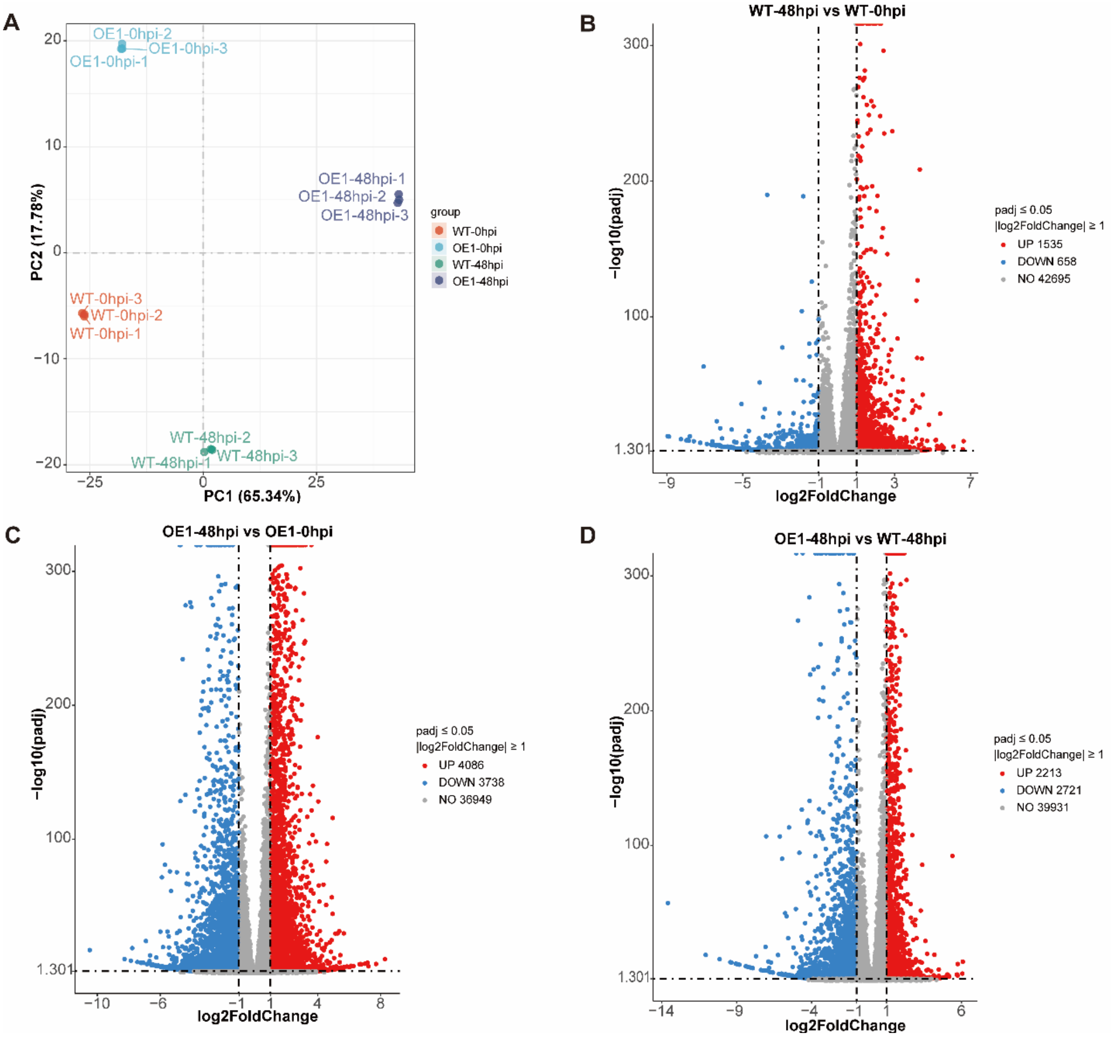

Figure S1. Transcriptome profiling and identification of differentially expressed genes in W82 and OE1 following *Phytophthora sojae* infection.

(A) Principal component analysis (PCA) of transcriptome data from W82 and OE1 under control (0 h) and 48 h post inoculation (48 hpi) conditions. Each point represents one biological replicate. **(B)** Volcano plot showing DEGs between W82 at 48 hpi and control. **(C)** Volcano plot showing DEGs between OE1 at 48 hpi and control. **(D)** Volcano plot showing differentially expressed genes (DEGs) between OE1 and W82 at 48 hpi. Red dots indicate upregulated genes, blue dots indicate downregulated genes, and gray dots indicate non-significant genes. Differential expression was defined as adjusted *p*-value (padj) < 0.05 and $|\log_2\text{FoldChange}| \geq 1$. Control indicates 0 h samples, and 48 hpi indicates samples collected 48 h after *P. sojae* inoculation.

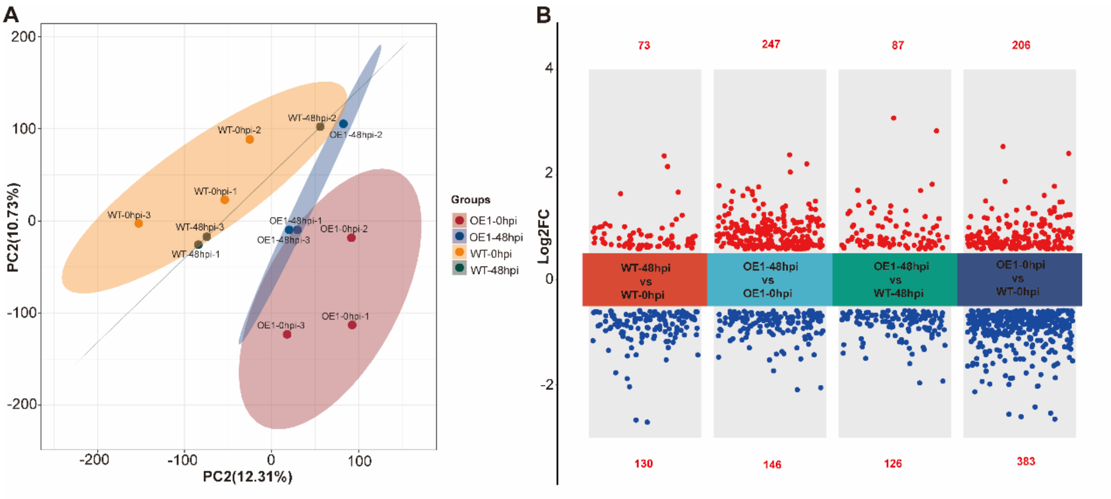

Figure S2. Global proteomic variation and differentially expressed proteins in OE1 and W82 after *Phytophthora sojae* inoculation.

(A) Principal component analysis (PCA) of protein abundance profiles in OE1 and W82 under control conditions and at 48 h post inoculation (48 hpi). Biological replicates showed close clustering, indicating good reproducibility of the proteomic data. **(B)** Volcano plots showing differentially expressed proteins (DEPs) in each comparison. Red and blue dots indicate upregulated and downregulated proteins, respectively.
